# Between heuristic and deliberative thinking: a multi-center qualitative study of physicians’ decision-making in infection prevention practice

**DOI:** 10.1186/s13756-025-01572-z

**Published:** 2025-05-15

**Authors:** Miriam Schutte, Mireille Dekker, Jonne Sikkens, Rosa van Mansfeld

**Affiliations:** 1 Department of Medical Microbiology and Infection Prevention, Amsterdam UMC, University of Amsterdam, De Boelelaan 1118, 1081 HV Amsterdam, The Netherlands; 2 Amsterdam Public Health Research Institute, Quality of Care, Amsterdam, The Netherlands; 3 Department of Medical Microbiology and Infection Prevention, Amsterdam UMC, Vrije Universiteit Amsterdam, Amsterdam, The Netherlands; 4 Amsterdam Institute for Infection and Immunity, Amsterdam, The Netherlands; 5 Department of Internal Medicine, Amsterdam UMC, Vrije Universiteit Amsterdam, Amsterdam, The Netherlands

**Keywords:** Physicians, Infection prevention, Decision-making, Interviews, Behavior, Theoretical domains framework

## Abstract

**Background:**

Application of standard infection prevention and control (IPC) measures is crucial to prevent hospital-acquired infections, but compliance by physicians is suboptimal. Interventions aimed to improve compliance are often generic and lack sustained effects. A better understanding of physicians’ trade-offs regarding application of IPC and influences on their behavior is needed to develop effective behavior change interventions. We aimed to understand physicians’ decision-making processes around application of IPC and the factors that influence their behavior.

**Methods:**

This qualitative study involved semi-structured interviews with 18 physicians and 7 nurses from five different hospitals in the Netherlands. Reflexive thematic analysis involved inductive coding followed by deductive analysis using mechanisms of action, including the Theoretical Domains Framework, that link to behavior change techniques.

**Results:**

We found heterogeneity in physicians’ approaches to decision-making around application of IPC. Some physicians relied on heuristics, while others applied logical reasoning. The latter group made an autonomous assessment of the risks for infection associated with a situation and traded off the costs and benefits of IPC application. The decision was further influenced by personal beliefs about the value of IPC and a supporting physical and social environment. Eighteen out of 26 mechanisms of action underlying the influences on IPC behavior were matched to our results; most important are “memory, attention and decision processes”, “behavioral cueing”, “beliefs about consequences”, “values”, “norms”, “social influences”, “social learning/imitation” and “environmental context and resources”. These findings suggest that interventions are most likely to be beneficial if these focus on developing heuristics, changing risk beliefs, using social norms and imitation and generating a supportive environment.

**Conclusion:**

The heterogeneity in physicians’ decision-making and autonomous risk assessment which is different from other healthcare professionals calls for tailored interventions targeting heuristic decision making, personal beliefs, social norms and the environmental context.

**Supplementary Information:**

The online version contains supplementary material available at 10.1186/s13756-025-01572-z.

## Introduction

Hospital-acquired infections are the most common complications of hospitalized patients and globally affect one in seven patients [1, 2]. Standard infection prevention and control (IPC) measures are crucial to prevent transmission of pathogens [3], and worldwide, up to 70% of hospital-acquired infections might be preventable [4]. However, compliance with IPC guidelines is suboptimal among physicians, who tend to lag behind compared to nurses [5, 6]. Interventions aimed to improve IPC compliance of healthcare professionals (HCPs) in general often lack sustained effects in physicians [5, 7].

Understanding the factors that influence behavior is critical to move towards effective interventions to improve compliance [8]. While factors related to nurses’ noncompliance have been explored frequently [9, 10], factors influencing physicians’ IPC behavior remain more elusive. Current literature reports that the local culture, availability of resources, role modeling, beliefs about consequences and time constraints are the most important influences on physicians’ IPC behavior [11]. These factors are however not unique to physicians, but also influence HCPs in general. To uncover why intervention effects for physicians are lacking while influencing factors among HCPs are similar, a deeper understanding of the mechanisms behind these factors is required.

The Theoretical Domains Framework is a commonly used framework to identify and categorize influences on behavior [12]. Recent advancements in behavior and implementation science have led to the introduction of ‘mechanisms of action’, which describe the processes through which behavior change occurs [13]. While factors influencing behavior might imply a linear relationship, concentrating on ‘what’ influences behavior, mechanisms of action emphasize the dynamic process by focusing on ‘how’ and ‘why’ interventions work to change behavior [14]. Building on the Theoretical Domains Framework, an ontology of 26 mechanisms of action was specified [15]. Through literature study and expert consensus, these mechanisms of action have been paired with behavior change techniques deemed most optimally suited to address these mechanisms underlying unwanted behavior [15, 16]. The term ‘mechanisms of action’ has been used before to describe interventions in various health areas, but with varying meanings [17]. The mechanisms of action ontology can help to unify the reporting of intervention development and evaluation. While a few examples of studies in which this ontology was applied have been published, its application has been limited thus far [18, 19]. In the field of IPC, the value of behavioral theory to underpin interventions has been recognized, but it has rarely been applied [20].

The aim of this study was to 1) identify factors influencing physicians’ IPC behavior and, 2) gain an in-depth understanding of physicians’ trade-offs in decision-making around IPC application. Taken together, we identified the mechanisms of action associated with the influencing factors to facilitate selection of suitable strategies for behavior change interventions.

## Methods

### Study design

This qualitative study involved semi-structured interviews with physicians and nurses from five Dutch hospitals. We followed the Consolidated Criteria for Reporting Qualitative Research [21] (Additional file 1).

### Participants and recruitment

To maximize variation in perspectives, we purposively recruited physicians from two university hospitals and three general hospitals with varying years of experience, specialisms and genders [22] (Table 1). During data collection, we sampled additional participants through snowball and opportunistic sampling. Twenty-seven invitations were sent to physicians by e-mail, with a follow-up sent one or two weeks later. Twenty physicians responded. All agreed to participate; two of these physicians were unavailable to participate after signing consent. During data analysis, participants were contacted again via e-mail to ask deepening questions through a phone call if additional questions arose. In addition, we included interviews with nurses to get an outsider perspective on physician behavior. Nurses from one university hospital and one general hospital were contacted via their team leaders or an infection prevention practitioner. Ten nurses responded, and three were not eligible because they did not work on general nursing wards. Seven agreed to participate. All participants were provided with an information sheet and asked to sign a consent form prior to participation.

**Table 1:** Sample characteristics

	**Physicians (*n* = 18) n (%)**	**Nurses (*n* = 7) n (%)**
*Gender*		
Female	9 (50)	7 (100)
*Setting*		
University hospital	14 (77.8)	6 (85.7)
General hospital	4 (22.2)	1 (14.3)
*Specialty*		
Medical	6 (33.3)	3 (42.9)
Surgical	6 (33.3)	1 (14.3)
Intensive care unit (ICU)	6 (33.3)	3 (42.9)
*Number of years of experience as medical specialist*		
< 0 (in training)	6 (33.3)	-
0–10	5 (27.8)	-
10–20	3 (16.7)	-
20 +	4 (22.2)	-

### Data collection

A researcher with a master’s level biomedical sciences background and trained in qualitative research (MS) conducted the semi-structured interviews. The interviewer had no existing relationships with the participants and limited knowledge of their context. Interviews were conducted at the workplace of the participant or online (Microsoft Teams, 2023) between October 2023 to May 2024 and lasted between 14 and 48 min. Additional phone calls lasted between 5 and 16 min. Only the interviewer and participant were present. Before the interview, the interviewer explained the purpose of the research.

Pilot-tested interview guides with open ended questions were used (Additional file 2). The interview guides were designed to maintain focus on thought processes and contextual influences in the real-world setting [23]. Physicians were asked to describe situations in which they did (not) apply IPC measures, elaborate on their trade-offs in this decision, and voice their needs for support. Nurses were asked to describe in which situations they saw physicians apply IPC or deviate from IPC guidelines, and what opportunities to support physicians they saw for themselves. Follow-up questions were asked to generate a deeper understanding of the participants’ perspective. Specific follow-up questions were iteratively added to the interview guides during analysis. Interviews were audio recorded and transcribed verbatim by MS. An audit trail, including field notes and reflexive memos to capture relevant contextual details, non-verbal expressions and initial thoughts after each interview, was kept. Interviews were held until data saturation was achieved; that is, when no new (nuances to) themes were identified from the data [22]. Two additional interviews were conducted to check data saturation.

### Data analysis

Analysis was conducted by researchers (MS, MD, RM, JS) experienced in qualitative research. The research team consisted of two full-time researchers, one clinical microbiologist and one internal medicine physician, with expertise in infection prevention, implementation science, epidemiology and psychology.

Interviews were analyzed using a reflexive thematic analysis within a contextualist theoretical approach [23, 24]. This approach focuses on understanding the data within its socio-cultural context and recognizes the researchers’ subjectivity as a resource in shaping the data. Interviews were discussed with the research group in regular meetings. Three researchers initially read the transcripts to familiarize themselves with the data and independently coded the first two transcripts (MS, MD, RM). The remaining transcripts were coded by two researchers (MS and MD or RM). Inductive, in vivo coding was applied to keep the codes close to participants’ wordings. Differences were discussed with all coders to foster collective understanding and reflect on differing interpretations. One researcher (MS) iteratively added all codes to a preliminary clustering into categories derived from the data to look for patterns. Shared meanings of categories were reviewed through discussions with the research group to refine categories and define overarching themes (MS, MD, JS, RM). Reflexive memos and transcripts were regularly consulted to maintain awareness of how the researchers’ perceptions might influence the findings and to ensure accuracy of interpretations with the data.

Mechanisms of action as defined in the Theory and Techniques Tool were fitted to the identified themes and subthemes independently by four researchers (MS, MD, RM, JS), followed by a group discussion to reach consensus [15, 16].

The final themes were described narratively, and their fit to the mechanisms of action was presented visually in a Sankey diagram. Key findings were supported with selected quotes in text; quotes were translated from Dutch to English.

MAXQDA 2022 for Windows (VERBI Software, 2021) was used to manage data coding [25]. R Version 4.3.2 in RStudio was used for data visualization using the networkD3 package for Sankey diagrams and htmlwidgets for figure lay-out customization [26–29].

### Ethics

A waiver for ethics approval was obtained from the Institutional Review Board at Amsterdam UMC (2023.0440).

## Results

### General characteristics

Eighteen physicians and seven nurses from two university hospitals and three general hospitals were interviewed through individual face-to-face or online interviews. Four additional phone calls were conducted to ask physicians deepening questions. After this, data saturation was considered to have been reached. The included physician population was diverse regarding experience, specialty and sex. All sample characteristics are shown in Table 1.

In the interviews, physicians from various specialties had distinct associations with IPC, which led to diverse topics of the interviews. Most physicians emphasized aspects such as isolation procedures, hand hygiene, and personal protective equipment. Other associations with IPC were disinfection and sterile practice. Perceptions about IPC varied across clinical settings. In an outpatient clinic, IPC was seen as part of routine procedures, while in an inpatient clinic or in the operating room, IPC required more attention and awareness. Surgeons, working both in the operating room and on the ward, mainly talked about IPC in the operating room. Overall, physicians noted that they aim to keep the risk of infection for the patient as low as possible.

We identified themes regarding heterogeneity in decision-making, autonomous risk assessment, trade-offs between costs and benefits, personal beliefs about the value of IPC and the need for cultivating a supportive work environment. These themes are described in detail below, followed by a visualization of the match between the themes and the mechanisms of action.

### Heterogeneity in decision-making

Participants’ approaches to deciding on application of IPC measures varied between heuristic and deliberative thinking. Heterogeneity in decision-making depended on physician characteristics, such as experience or specialty, and the context, such as the operating room or outpatient clinic, patient characteristics, type of task or urgency of the situation. Some physicians described a type of decision-making in which they relied on guidelines, habits or heuristics. For example, several medical specialists in training expressed to automatically follow what they learned in the workplace from supervisors or what was stated in guidelines. *“Well, there are of course guidelines, right, for certain infections (…). It’s not that I personally handle that [certain infections] differently, I just follow the guidelines.” (ICU physician 4, in training).* Senior physicians employed more experience-based heuristics. Another more deliberate type of decision-making was described based on logic and reasoning. These participants, mainly senior physicians, described an autonomous risk assessment to determine whether IPC application would be of added value. *“Sometimes you can deviate from a guideline because in that situation you don’t see the added value as a medical specialist ultimately responsible for the patient. (…) You can deviate from any protocol, provided it is substantiated, and ultimately the specialist carries the responsibility.” (Surgeon 1).*

### Autonomous risk assessment

When an autonomous risk assessment was used, physicians described basing this decision on logical reasoning and common sense rather than guidelines. They explained that while they might not know all IPC guidelines by heart, they felt that their knowledge formed by previous experiences and education was sufficient to estimate the infection risks and decide on the application of IPC.

To judge the risks for infection in a situation, physicians considered the type of task (physical contact, working with wounds or bodily fluids), patient characteristics (vulnerability, type of infection) and setting (operating room, ward, ICU). In some settings, such as the ICU and the operating room, physicians expressed to be extra aware of the risks for infection. At the point of care, physicians considered whether they had touched the patient or their surroundings and whether it was visibly soiled. Physicians described these considerations to be overruled by urgency in acute situations. In emergencies, IPC was deemed less important and other priorities prevailed. *“In emergency surgeries, the flow is very different, and steps are skipped to save a life. And if that means the risk of infection is potentially slightly higher, I accept that.” (Surgeon 1). “I have a clear image of what is important at that [acute] moment and that is not IPC measures.” (ICU physician 3).*

### Trade-offs between costs and benefits

In addition to a risk assessment, physicians considered the costs and benefits of IPC application based on various factors, including environmental sustainability, social norms, effort, and practical feasibility.

#### Environmental sustainability

One physician described the tension between IPC and sustainability: *“Where formerly it was primarily about doing everything to potentially prevent as much as possible, now there is a shift towards: it needs to be proven to be useful before implementing things. This also has to do with (…) sustainability, with global warming, CO2, nitrogen, all those things, making clinicians think more about the added value of an intervention.” (Surgeon 1).* Arguments about sustainability were largely made by female participants.

#### Social norms

Several physicians recognized a social norm on their ward. Some physicians described the norm to be set by all colleagues on the ward, while others mentioned a considerable influence from the head of the department. A trade-off was described between wanting to adhere to the norm, to fit in with the rest, and to act on your own beliefs. Some physicians sometimes felt like they were alone in stimulating IPC application and were demotivated from trying to convince their colleagues. Others expressed to feel a shared responsibility for IPC application on the ward.

#### Effort

The feeling among some physicians was that the more effort it takes to apply IPC, the less inclined a physician is to do so. *“Everyone always thinks it is a bit difficult, right? IPC. (…) It requires something extra anyway.” (ICU physician 2).*

#### Practical feasibility

Several physicians reported to struggle with practical feasibility of IPC guidelines. They explained that guidelines are often too theoretical, without much consideration of the limitations of the practical reality, leading to frustration and irritation. *“From an infection prevention standpoint, I can understand that every unwanted spread is one too many, but from a care operations standpoint, I sometimes wonder if it's a bad thing if we accidentally place someone on a ward once without proper precautions. That can be hard for me at times because the stricter we are about infection protection, the less manageable care operations become.” (Internal medicine physician 2).* Several, mainly senior and ICU, physicians experienced logistical barriers, for instance not having enough beds or single rooms available for isolation. One physician explained: *“The ward is full, we have a limited number of single-person rooms and only two real isolation rooms, so yes, if these rooms aren’t available, it [isolation] just stops.” (ICU physician 1).*

### Personal beliefs about the value of IPC

Physicians’ decision-making on IPC application was further influenced by personal beliefs about the value of IPC. Several physicians stressed the importance of awareness for IPC, which was described as recognizing the value of IPC, consciously paying attention to it, and understanding the risks and consequences of their IPC behavior. Some physicians said to feel the urgency of IPC because they experienced an outbreak or work with a vulnerable patient population. Several physicians voiced that the coronavirus disease 2019 (COVID-19) pandemic contributed to a (temporary) increase in attention for IPC. *“In that COVID time of course we had to wear a face mask and gloves et cetera, then we were doing it [IPC application] very consciously. (…) I know it [IPC application] became much better, since the COVID time it [IPC] received much more attention, but it also declines again, so maybe it is good to still have attention for it [IPC].” (Gynecologist 1).*

Despite its recognized importance, physicians stated that attention for IPC is lacking. Several physicians described that not deliberately thinking about IPC or acting out of habit led to forgetting IPC application. *“Well, I always just call it a lack of interest or awareness, just that you think like, I just go and do my rounds or something. I don’t know, just that you don’t consciously think about it [IPC].” (Hospital physician 1).* Nurses also observed this lack of attention. From their perspective, physicians do not seem to realize the consequences of noncompliance, such as an outbreak or isolation. Nurses who spend the most time at the bedside felt like they are impacted more by these consequences than physicians who come and go. Nurses noted that especially consultants did not adequately apply IPC measures on their wards. Several physicians also voiced this concern. *“Consultants, just without any form of IPC measures, touch a patient and this is not addressed, that gives some frustration sometimes.” (ICU physician 2).*

Beliefs about the value of IPC were also based on the available evidence. For example, evidence about the effectiveness of an IPC measure convinces physicians of its usefulness. Only men, primarily senior physicians and surgeons mentioned the importance of evidence.

### Needs: Cultivating a supportive work environment

In addition to the above, physicians articulated several needs for IPC application. These included a supportive physical and social environment, specifically a positive ward culture and a close relationship with the IPC team.

#### Physical environment

Physicians described that a facilitating environment, with sufficient availability of and access to resources such as hand disinfectant, helps to adequately apply IPC. Most physicians stated that materials such as gloves and gowns are readily available, while some noted empty hand disinfectant dispensers to be a barrier. Physicians named that it would help them if it was easier to apply IPC. Some suggested succinct, visual versions of protocols to be put on the walls and serve as reminders or streamlining and automating processes such as isolation labelling.

#### Social environment: ward culture

Several physicians recognized their own influence as a role model. *“Another important consideration is that you’re supposed to be a role model. I think then you always need to do it [apply IPC]. (…) I think that out of routine, to set the example, good behavior will be followed, and so I should do it [apply IPC].” (Internal medicine physician 2)*. One physician described peers also learn from each other, for example during rounds of the ward.

Most physicians stated that they would alert a colleague on noncompliance if they saw it and are open to receiving feedback on their own behavior. Several physicians described that this is possible in a culture where people show understanding, offer constructive feedback and a dialogue and everyone recognizes the importance of IP. Hierarchy was named to inhibit addressing noncompliance of colleagues.

Nurses explained they sometimes alerted physicians when they deviated from guidelines, but the perceived ease of approaching physicians differed per ward and per person. From nurses’ perspective, it helped if the physician was a standard staff member with an approachable demeanor. However, nurses did not perceive alerting physicians on noncompliance as their responsibility. Nurses suggested to introduce repeated moments of IPC education and add IPC to introductions for new physicians. Nurses felt that they could contribute to this by giving clinical lessons and by engaging physicians more in the recurring IPC awareness days that nurses already have among themselves.

#### Social environment: Physicians’ expectations of the IPC team

Several physicians expressed the need for clear guidelines and communication about these by the IPC team. Mostly physicians from medical specialties found it unclear what guidelines were in place and where to find these. In line with this feeling, nurses experienced that physicians regularly ask them what to do because they do not know the guidelines.

Both physicians and nurses expressed that attention for IPC should be raised regularly, and that the IPC team should be proactive in this. For example, attention can be increased through campaigns, quality improvement projects, visual reminders and feedback. Regarding feedback, physicians described that insight into the numbers of infections, rather than for instance the hand hygiene compliance numbers, would increase their attention for IPC.

A desire for the IPC team to visit the ward more often was voiced by several physicians. Physicians felt that regular visits would foster a closer relationship and facilitate better communication, understanding and collaboration. Nonetheless, these physicians expressed they don’t want to be too involved themselves but envision a more active role for nurses. *“What the ideal relationship [with the IPC team] would look like, well maybe not through me, but through the supporting roles in the hospital. For example, we have nurses with IPC as focus topic and this nurse collaborates with the IPC team (…) and then I can occasionally give input like, hey, this is important for physicians specifically (…). But especially that I am not always involved in that contact.” (Gynecologist 1).*

### Identified themes and matching mechanisms of action

**Fig. 1:**
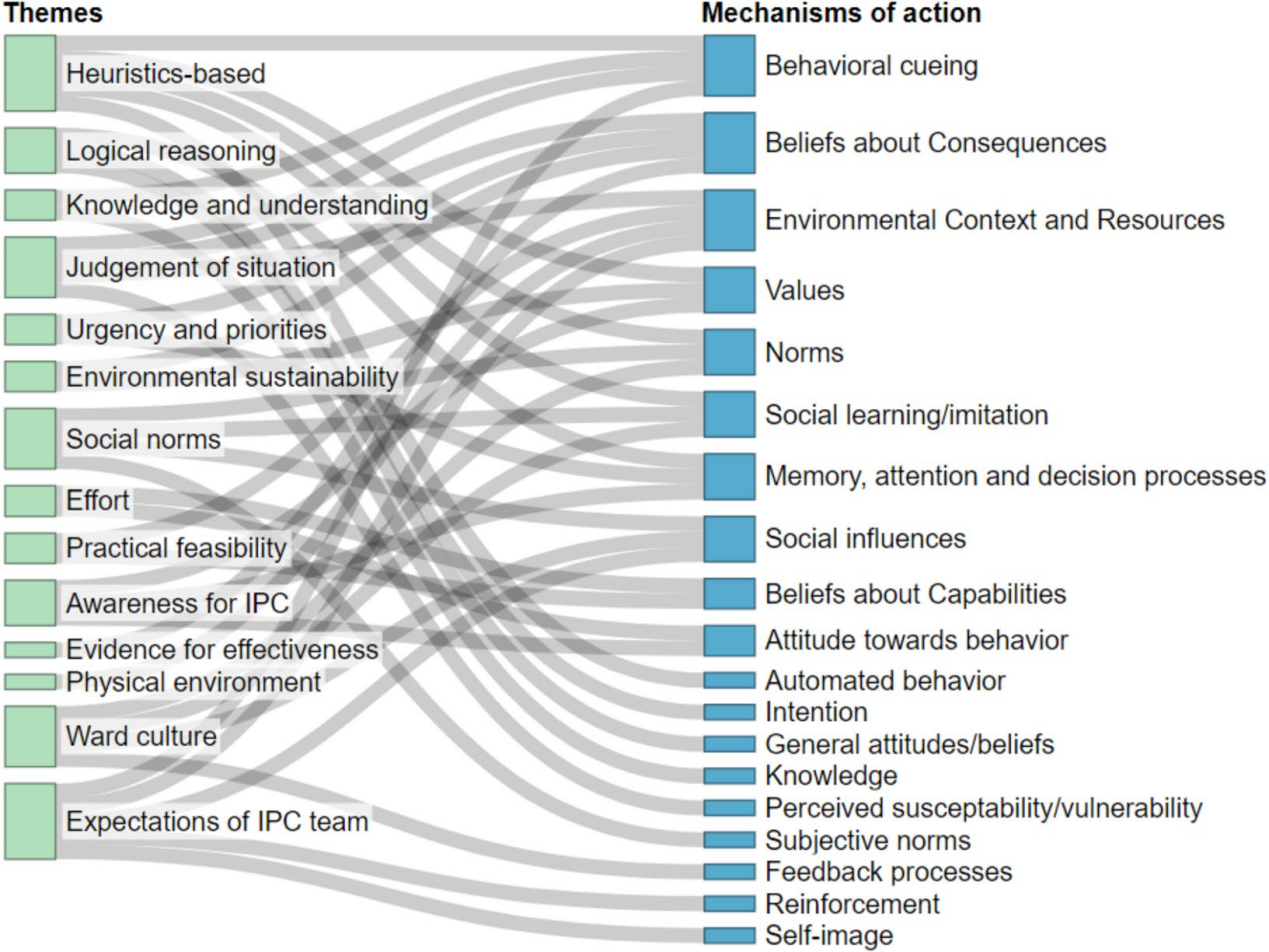
Sankey diagram of the match between interview themes and subthemes and mechanisms of action. The left nodes (green) represent the identified themes, presented in the order in which they are discussed. Through the grey links, they are connected to the mechanisms of action (blue) that were matched to each theme. Mechanisms are ordered with the mostly linked mechanisms on top

The identified themes of influences on physician’s IPC behavior described above highlight heterogeneity, dependance on personal beliefs and contextual influences. To enable linking these influences to theory-based targets for interventions, themes were matched to mechanisms of action (Fig. 1 and Additional file 3). Eighteen out of 26 mechanisms of action of the Theory and Techniques Tool were matched to our results; “automated behavior” was added since no mechanism fit. The identified mechanisms of action with a high potential as targets for interventions include “behavioral cueing”, “beliefs about consequences”, “environmental context and resources”, “values”, “norms”, “social learning/imitation”, “memory, attention and decision processes”, and “social influences”, depending on the specific context. To stimulate heuristic decision-making, “memory, attention and decision processes” and “behavioral cueing” can be targeted to develop mental cues and form habits. Targeting “beliefs about consequences” can guide towards making an adequate judgement and increase awareness for IPC. Regarding “environmental context and resources”, the context can both influence the risk assessment, and environmental cues can support heuristic thinking. Mechanisms involving social aspects such as norms, social influences and imitation are relevant across multiple themes, highlighting the potential impact of targeting these mechanisms. By targeting both these social aspects and the environmental context, interventions can address the need for generating a supportive work environment.

## Discussion

This qualitative study uncovered heterogeneity in the decision-making process of hospital physicians regarding their application of IPC. Some decision-making is based on heuristics or habits, while another type relies on logical reasoning. In the latter, physicians assess the risks associated with a situation and trade-off various factors such as feasibility and sustainability. Their behavior is further influenced by their personal beliefs about the value of IPC and external influences, being social support and their physical environment.

Autonomous risk assessment and deliberate reasoning seemed to be employed mostly by senior physicians in this study. Junior physicians appeared to rely more on heuristics, guidelines and behavior of supervisors. We hypothesize a shift towards more deliberate reasoning as physicians gain experience, and development of experience-based heuristics. Our results suggest junior physicians might be more affected by external influences, such as behavior of others, and this shifts more toward internal influences, such as personal beliefs, over time. In line with this view, Gilbert and Kerridge noted that medical students are taught about IPC, but as trained physicians, they take over the habituated behaviors of their seniors [30]. Shah et al. found that personal experience shapes HCPs risk evaluations [31].

While the factors that influence physicians’ IPC behavior are similar to those relevant to other HCPs [5], the autonomous risk assessment seems more prominent among physicians. Accordingly, most physicians in the qualitative study by Squires et al. expressed that hand hygiene requires a conscious decision [32]. A study among nurses, in contrast, found hand hygiene to largely be an automatic process [33]. In the current study, nurses spoke in terms of knowledge about rules and that physicians don’t know these, while physicians talked more about logic and reasoning. Similarly, McDonald et al*.* noted that physicians value unwritten rules and socially accepted behaviors more than written rules, while nurses stick to written rules and see noncompliance as unprofessional [34]. Physicians tend to be critical of guidelines in general [35–37]. These professional differences support the notion that interventions targeting nurses or physicians should address different mechanisms of action.

The relevant mechanisms of action we identified in this study link to behavior change techniques, facilitating selection of strategies for interventions [15, 16]. Our results suggest that interventions to improve physicians’ IPC compliance should focus on developing adequate heuristics to guide the risk assessment, changing risk beliefs, providing social support and making it easier to apply IPC. Use of the Theory and Techniques Tool, which presents links between mechanisms and strategies, aids the selection of strategies that are likely beneficial to target these mechanisms [16]. We describe our suggestions below.

Physicians develop heuristics which makes decision-making easier and more efficient, alleviating the need to remember all guidelines. Classic educational strategies to improve knowledge might therefore not be exhaustive to support physicians. Rather, such strategies should be supplemented with strategies to help physicians develop adequate heuristics, e.g., by providing cues and stimulating action planning. Cues can provide a stimulus to perform a behavior. Action planning links a cue to a behavioral response by forming ‘if–then’ plans specifying when and where the behavior will be performed [38]. For example, an ‘if–then’ plan could be: “if I enter a patient room, then I disinfect my hands”. This strategy might help to translate knowledge into actions in the work environment.

Physicians in this study expressed the need for social support both by colleagues on the ward and the IPC team. However, they did not envision a significant role for physicians themselves in the contact with the IPC team. Since physicians learn from and imitate other physicians’ behavior, especially from senior colleagues who function as role models, the use of trusted sources as champions might be effective. Champions, for instance an esteemed colleague or the team manager, can spread critical IPC information and provide real-time feedback [39]. Proposed mechanisms of using clinical champions are creating behavioral intentions through peer buy-in and modelling, and actual application through increasing skills and providing mentorship [39]. When IPC behaviors are endorsed by their peers rather than external teams, physicians are more likely to follow their example. The continuous presence of champions could foster sustained IPC compliance.

Based on our suggestions described above, a multimodal intervention to improve physicians’ IPC behavior could combine behavior change techniques such as action planning, cues, social support, identification of self as role model, a credible source, information about consequences, and feedback on behavior [15]. To ensure that a strategy not only addresses a factor of influence, but also the relevant mechanism, local teams should determine how generic strategies such as action planning, cues, champions and feedback could be operationalized to be most effective in their specific context. For example, Smiddy et al. saw that physicians are more sensitive to personalized feedback than group-level feedback [40].

### Strengths and limitations

A strength of this study is its qualitative design to allow in-depth exploration of various perspectives. Inclusion of physicians and nurses enhanced the breadth of the data. Our comprehensive and structured analysis, triangulating individual interpretations of research team members including a physician working in direct patient care, added methodological rigor. Presentation of links between themes and mechanisms of action adds a theoretical layer, facilitates translation of the results into implementation strategies and enables easier comparison to findings of others.

Our study has its limitations. Voluntary participation could have led to a bias towards participants with an affinity for IPC. However, since respondents expressed both negative and positive views, we anticipate limited impact of this bias on the identified themes and mechanisms of action. Different settings, specialties and levels faced distinct IPC settings and challenges, such as the operating room versus the ward or hand hygiene versus isolation precautions. While this adds complexity and diminishes the transferability, it showcases the breadth and depth that was desired by the qualitative approach. Nonetheless, we expect our findings to be sufficiently transferable to various physicians working in different hospital settings. It should be noted that observation of participants’ IPC behavior was beyond the scope of this qualitative study, and therefore the perceptions that were expressed do not directly translate to IPC compliance in practice. Finally, quotes have been translated from Dutch to English, whereby some meaning might have gone lost in translation.

## Conclusions

Our findings demonstrated heterogeneity among physicians in their decision-making process for applying IPC. Depending on personal attributes of the physician and contextual characteristics, physicians make decisions based on heuristics or apply logical reasoning to weigh cost and benefits of specific measures. The diversity of settings and challenges that physicians encounter calls for interventions tailored to physicians, and preferably to relevant physician subgroups (e.g., junior vs. senior). To fit with mechanisms of action relevant for physicians, interventions should focus on developing heuristics, changing risk beliefs and providing a supportive environment.

## Supplementary Information

Additional file 1. COREQ checklist. This file contains the filled in COREQ checklist for this manuscript.

Additional file 2. Interview guides. This file contains the interview guides used for physicians and nurses.

Additional file 3. Matching themes and mechanisms. This file contains a table including all mechanisms of action that were matched to our identified themes.

## Data Availability

No datasets were generated or analysed during the current study.

## Abbreviations

COVID-19: Coronavirus disease 2019
HCP: Healthcare professional
ICU: Intensive care unit
IPC: Infection prevention and control
