# Supplementary material for: Between heuristic and deliberative thinking: a multi-center qualitative study of physicians’ decision-making in infection prevention practice
Source: Antimicrob Resist Infect Control. 2025 May 15;14:50. doi: 10.1186/s13756-025-01572-z (PMC12082995; doi:10.1186/s13756-025-01572-z)
Supplement: Supplementary file 2 — Additional file 2. Interview guides. This file contains the interview guides used for physicians and nurses. [file 13756_2025_1572_MOESM2_ESM.docx]

Supplementary material 2

Supplementary table 1: Semi-structured interview guide for physicians.

| Introduction and context | - Could you introduce yourself? (years of work experience, occupation) - Can you name examples of aspects related to IPC that you apply in daily practice? - Are you familiar with the IP team in the hospital and what they do? |
| --- | --- |
| Main questions |  |
| Application of IP | - When do you apply the IPC measures that you just described? - What considerations do you make to apply these measures? |
| Deviation from IPC guidelines | - When do you not apply these IPC measures? - What considerations do you make to not apply these measures? |
| Barriers and facilitators to compliance | - What currently helps you to apply IP in accordance with guidelines? What makes it easier? - What are obstacles that you encounter to apply IP? What makes it difficult? - Are there any other influences on your decision to apply IP? |
| Needs for support | - In the ideal situation, what could hypothetically help you to apply IPC? What do you need for this? |
| Specific follow-up questions | - How does [the factor that is mentioned] influence your application of IP? - [Surgeon] – Is your application of IPC different in the OR versus on the ward? - [Point of improvement] – What needs to change for you? What is required for this? How can the IP team help? - [Feasibility] – What makes it not feasible? What do you do in that situation? Is there an alternative? - [Contact with IP team] – What should the ideal relationship look like? What are the roles of the physician and the nurse in this? - [Addressing noncompliance] – When do you address noncompliance of colleagues? What is the culture like? How does it affect your behavior if someone alerts you on your noncompliance? |
| Conclusion | - Are there any things we have not discussed that you would like to mention about this topic? |

Supplementary table 2: Semi-structured interview guide for nurses.

| Introduction and context | - Could you introduce yourself? (department of work) - Can you name examples of aspects related to IPC that you apply in daily practice? |
| --- | --- |
| Main questions |  |
| Application of IP by physicians and deviation from guidelines | - When do you see physicians apply IPC measures according to the guidelines? - When do you see physicians deviate from IPC guidelines? - Is the compliance with IPC guidelines of physicians different for certain IPC measures compared to others? |
| Contextual influences | - In what contexts does IP application/deviation from guidelines occur? What makes IPC application easier/difficult for them? - What could influence this behavior? |
| Nurses’ role in supporting physicians | - [For infection control link nurses] – Do you see any effects of infection control link nurses on physicians? Should this change? - What do you personally do to support physicians’ IPC application? What role do you see for nurses to support physicians? - If you see noncompliance of physicians, do you address this? What is the culture like? |
| Opportunities for support | - What could help physicians to better apply IPC measures? What would be required for this? |
| Specific follow-up questions | - How does [the factor that is mentioned] influence physicians’ application of IP? - Can you name an example specifically for physicians’ IPC application? - How are physicians being engaged in [IPC activity among nurses that is mentioned]? Should this change? - [Point of improvement] – What needs to change for you? What is required for this? How can the IP team help? |
| Conclusion | - Are there any things we have not discussed that you would like to mention about this topic? |
