# Supplementary material for: Between heuristic and deliberative thinking: a multi-center qualitative study of physicians’ decision-making in infection prevention practice
Source: Antimicrob Resist Infect Control. 2025 May 15;14:50. doi: 10.1186/s13756-025-01572-z (PMC12082995; doi:10.1186/s13756-025-01572-z)
Supplement: Supplementary file 3 — Additional file 3. Matching themes and mechanisms. This file contains a table including all mechanisms of action that were matched to our identified themes. [file 13756_2025_1572_MOESM3_ESM.docx]

Supplementary material 3

Supplementary table 3: Match between interview themes and subthemes and mechanisms of action.

| Themes and subthemes | Mechanisms of action |
| --- | --- |
| *Heterogeneity in decision-making* |  |
| Heuristics-based | Norms  Values  Behavioral cueing  Social learning/imitation  Automated behavior* |
| Following the guidelines | Norms  Values  Behavioral cueing |
| (Practical) training and supervision | Social learning/imitation |
| Logical reasoning | Memory, attention and decision processes  Intention  General attitudes/beliefs |
| *Autonomous risk assessment* |  |
| Knowledge and understanding | Knowledge  Behavioral cueing |
| Judgement of situation | Beliefs about consequences |
| Setting | Beliefs about consequences  Environmental context and resources |
| Type of activity | Beliefs about consequences  Perceived susceptibility/vulnerability |
| Patient characteristics | Beliefs about consequences  Perceived susceptibility/vulnerability  Behavioral cueing |
| Urgency and priorities | Beliefs about consequences  Memory, attention and decision processes |
| *Trade-offs between costs and benefits* |  |
| Environmental sustainability | Beliefs about consequences  Values |
| Social norms | Norms  Social learning  Social influences |
| Shared responsibility | Subjective norms |
| Effort | Beliefs about capabilities  Attitude towards the behavior |
| Practical feasibility | Environmental context and resources  Beliefs about capabilities |
| *Personal beliefs about value of IPC* |  |
| Awareness for IPC | Memory, attention and decision processes  Values  Attitude towards the behavior |
| Lack of attention for IPC | Memory, attention and decision processes  Values  Attitude towards the behavior |
| Evidence for effectiveness of IPC measures | Beliefs about consequences |
| *Needs: Cultivating a supportive work environment* |  |
| Physical environment |  |
| Resources and materials | Environmental context and resources |
| Ward culture |  |
| Role modelling | Social learning/imitation  Social influences |
| Addressing noncompliance of colleague | Feedback processes  Norms |
| Physicians’ expectations of the IPC team |  |
| Communication about guidelines by IPC team | Environmental context and resources  Reinforcement  Social influences |
| Efforts to increase attention for IPC | Reinforcement  Behavioral cueing  Environmental context and resources |
| Visibility of IPC team on the ward | Reinforcement  Social influences |
| Expectations about relationship with IPC team | Social influences  Self-image |

**’Automated behavior’ was added as an additional mechanism of action, since no mechanism of action captured this aspect in our view.*
